# White matter integrity and pro-inflammatory cytokines as predictors of antidepressant response in MDD

**DOI:** 10.1192/j.eurpsy.2022.1415

**Published:** 2022-09-01

**Authors:** S. Breit, E. Mazza, S. Poletti, F. Benedetti

**Affiliations:** 1 University Hospital of Psychiatry and Psychotherapy of Bern, University Hospital Of Psychiatry And Psychotherapy, Bern, Switzerland; 2 IRCCS Ospedale San Raffaele, Milano, Psychiatry And Clinical Psychobiology/neuroscience, Milan, Italy

**Keywords:** white matter integrity, biomarkers, MDD, pro-inflammatory cytokines

## Abstract

**Introduction:**

Major depressive disorder (MDD) often involves immune dysregulation with high peripheral levels of pro-inflammatory cytokines that might have an impact on the clinical course and treatment response. Moreover, MDD patients show brain volume changes and white matter (WM) alterations that are already existing in the early stage of illness.

**Objectives:**

The aim of the present review is to elucidate the association between inflammation and WM integrity and its impact on the pathophysiology and progression of MDD as well as the role of possible novel biomarkers of treatment response to improve MDD prevention and treatment strategies.

**Methods:**

We conducted an electronic literature search of PubMed on studies that examined the role of inflammation in depression and that focused on WM integrity and pro-inflammatory cytokines as predictors of antidepressant response.

**Results:**

There is evidence for central effects of peripheral inflammation which could activate microglia which, in turn, might trigger a cascade of inflammatory processes leading to neurotransmitter imbalances. Numerous studies indicated that both altered levels of peripheral inflammatory markers, particularly TNF-α, IL-6, and CRP as well as WM integrity might predict antidepressant treatment outcome.

**Conclusions:**

Despite mounting evidence on the impact of the immune system on WM microstructure, no study has yet addressed the interaction between the two factors in influencing antidepressant response. There is a lack of reproducible biomarkers predicting treatment response on an individual basis. The availability of such biomarkers would enable more efficient and personalized treatments with a faster treatment response and better prevention of treatment resistance.

**Disclosure:**

No significant relationships.

